# Supplementary material for: Glucose Oligosaccharide and Long-Chain Glucomannan Feed Additives Induce Enhanced Activation of Intraepithelial NK Cells and Relative Abundance of Commensal Lactic Acid Bacteria in Broiler Chickens
Source: Vet Sci. 2021 Jun 12;8(6):110. doi: 10.3390/vetsci8060110 (PMC8231533; doi:10.3390/vetsci8060110)
Supplement: Supplementary file 1 [file vetsci-08-00110-s001.zip › vetsci-1240193-supplementary.pdf]

Supplementary Table S1. Clarification of compounds used in the in vitro screening.

Group	Code	Compound
Plant extract	PE1	Andrographolide[1]
	PE2	Apigenin[1]
	PE3	*Astragalus Membranaceus*[2]
	PE4	Garlic extract 1[2]
	PE5	Garlic oil[1]
	PE6	Luteolin[1]
	PE7	Resveratrol[1]
	PE8	*Andrographis paniculata*[1]
	PE9	Tocopherol (blend; delta-, beta-, gamma, alpha alpha-tocopherol)[3]
	PE10	Curcuma longa[3]
	PE11	Polyphenols/Antioxidants[3]
	PE12	Onion extract[3]
	PE13	Grape extract[3]
	PE14	Rosmaniric acid[3]
	PE15	Thymol[3]
	PE16	Citral[3]
	PE17	Geraniol[3]
	PE18	Garlic extract 2[3]
	PE19	Eugenol[3]
	PE20	Para-cymene[3]
	PE21	Cinnamic aldehyde[3]
	PE22	Carvacrol[3]
	PE23	Plant extract blend of zinc and selenium[3]
Fermentation	FP1	2,3-Butanediol[1]
	FP2	2-Ketogulonic acid[3]
Vitamin	V1	β-Carotene[1]
	V2	Retinoic acid[1]
Drug	D1	Lenalidomide[1]
	D2	Pomalidomide[1]
Lipid	L1	Linoleic acid, conjugated[1]
	L2	Medium-chain fatty acids[3]
	L3	Mannosylerythritol lipids[3]
Fungus extract	FE1	Shiitake mushroom[2]
	FE2	*Tremella fuciformis*[2]
Polysaccharide	P1	Glucose oligosaccharide[3]
	P2	Glucomannan – long chain[3]
	P3	Polydextrose[3]
	P4	Resistant dextrin[3]
	P5	Glucomannan – short chain[3]
	P6	Glucomannan – medium chain[3]
	P7	Scleroglucan[3]
	P8	Glucan[3]
	P9	Algae (whole product) - *Euglena gracilis*[3]
	P10	Algae extract - *Euglena gracilis*[3]
	P11	Algae (whole product) - *Ulva lactuca*[3]
	P12	Algae extract - *Ulva lactuca*[3]

	P13	Algae extract - *Laminaria sp*. and/or *Ascophyllum nodosum*[3]
	P14	Yeast cell wall 1 - *Saccharomyces cerevisiae*[3]
	P15	Yeast cell wall 2 - *Saccharomyces cerevisiae*[3]
	P16	Yeast cell wall 3 - *Saccharomyces cerevisiae*[3]
	P17	Algae insoluble polysaccharides[3]
	P18	Chitosan[3]
	P19	Chitosan oligosaccharide[3]
Acid/Salt	AS1	Calcium butyrate 95%[3]
Blend	B1	Blend of essential oils and organic acids[3]
Yeast	Y1	Yeast culture - *Saccharomyces cerevisiae*[3]
Modified sugar	MS1	Alkyl glycoside - 83.5%[3]
	MS2	Alkyl glycoside - mixture[3]
	MS3	Alkyl glycoside - 95%[3]
	MS4	Oligodextran[3]
	MS5	Glucuronolactone[3]
	MS6	Octyl-β-D-glucoside[3]
Simple sugar	SS1	Erythrulose[3]
	SS2	Keto-sugar[3]
Emulsifier	E1	Sugar ester (100% di-, tri-, polyester) [3]
	E2	Sugar ester (50% mono-, 50% di-, tri-, polyester) [3]
	E3	Citrilated mono-diglycerides[3]
	E4	Sorbitan monostearate 1[3]
	E5	Sorbitan monostearate 2[3]

[1]Sigma-Aldrich, the Netherlands, [2]Shaanxi Yuwangtang Biotechnology Development Co. Ltd., China, [3]Cargill Inc., USA.

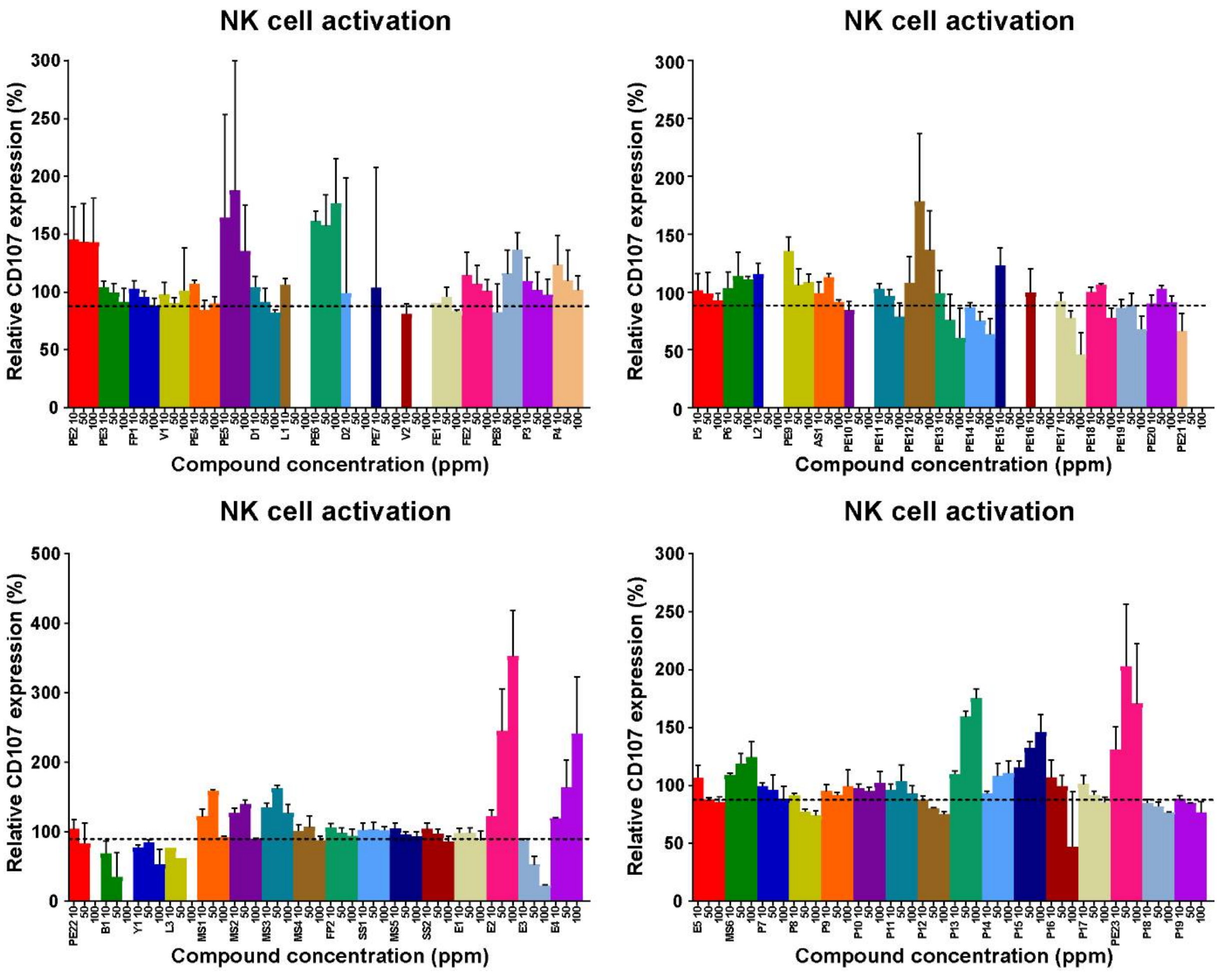

Supplementary Figure S1. In vitro screening of feed compounds for their effect on NK cell activation. NK cell activation was measured by expression of CD107 on NK cells (%) and CD107 expression is expressed relative to the negative control, which was set at 100%. Mean + SEM is shown (n = 3) of the compounds plant extracts (PE), fermentation products (FP), vitamins (V), drugs (D), lipids (L), fungus extracts (FE), polysaccharides (P), acid/salts (AS), blend of essential oils and organic acids (B), yeasts (Y), modified sugars (MS), simple sugars (SS) and emulsifiers (E) and dotted line represents level of the solvent control.

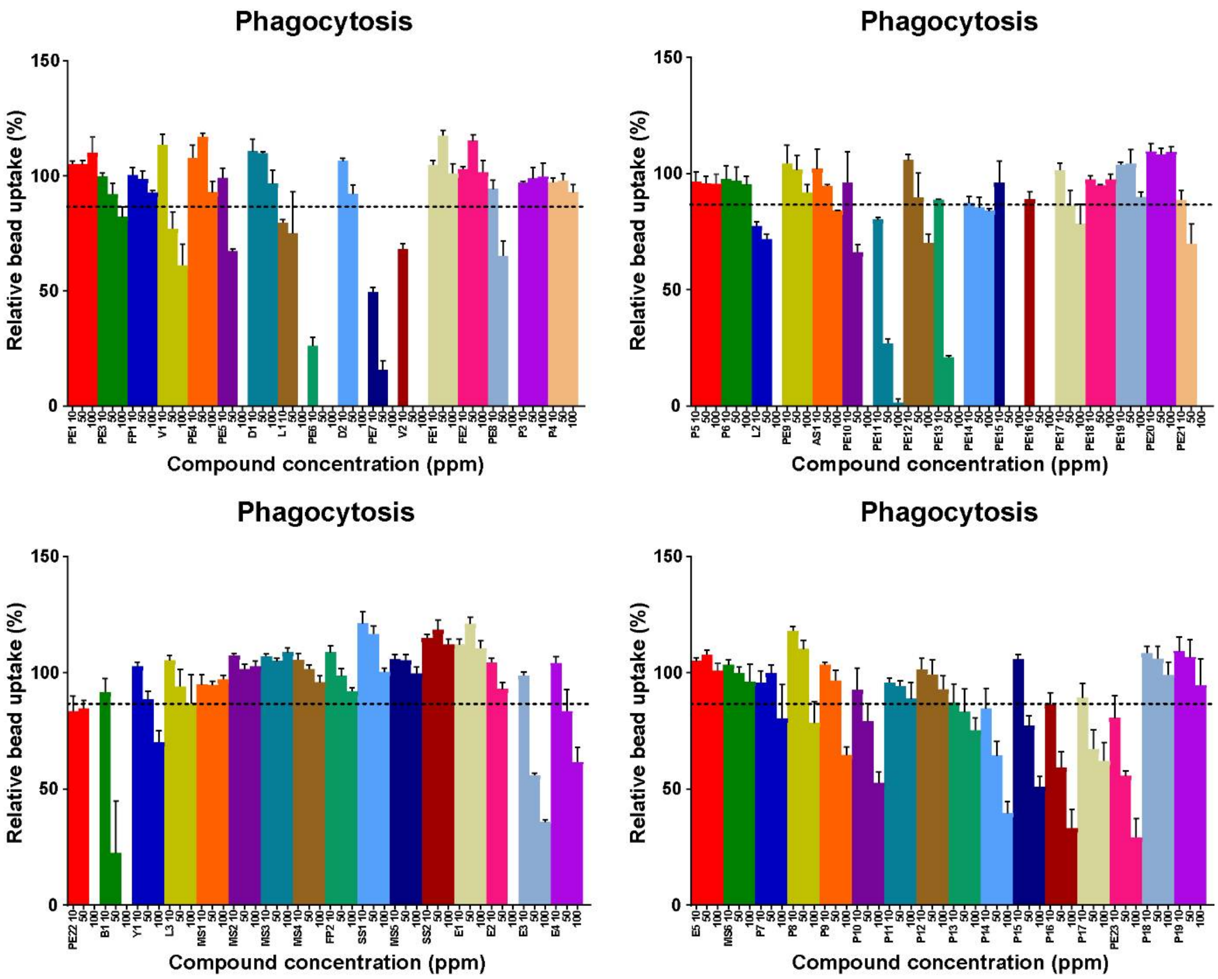

Supplementary Figure S2. In vitro screening of feed compounds for their effect on phagocytosis of macrophages. Phagocytosis of macrophage-like HD11 cells (%) is expressed relative to the reference control which was set at 100%. Mean + SEM is shown ($n = 3$) of the compounds plant extracts (PE), fermentation products (FP), vitamins (V), drugs (D), lipids (L), fungus extracts (FE), polysaccharides (P), acid/salts (AS), blend of essential oils and organic acids (B), yeasts (Y), modified sugars (MS), simple sugars (SS) and emulsifiers (E) and dotted line represents level of the solvent control.

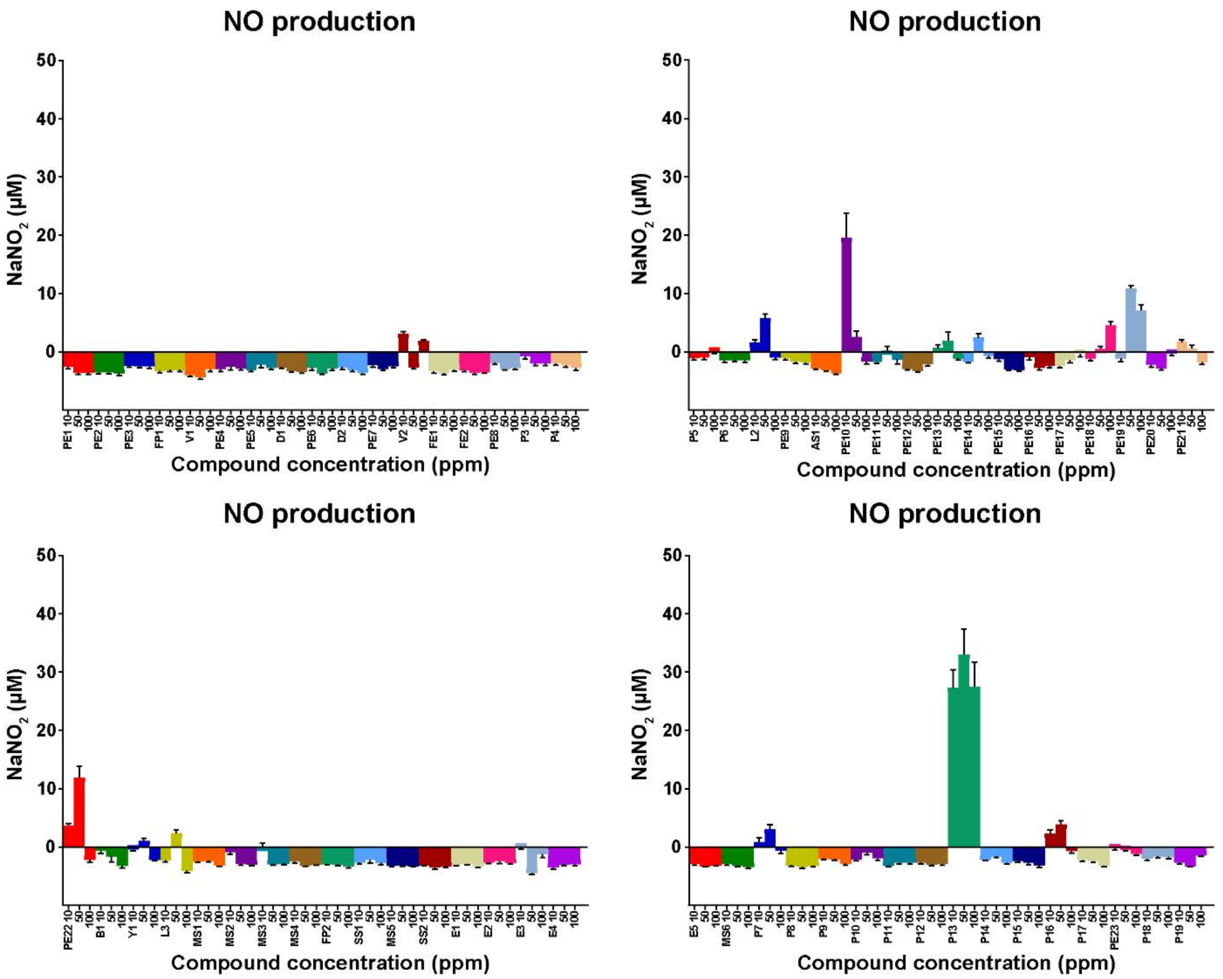

Supplementary Figure S3. In vitro screening of feed compounds for their effect on NO production of macrophages. NO production of macrophage-like HD11 cells was assessed by measuring nitrite concentration (μM). Mean + SEM is shown (*n* = 9) of the compounds plant extracts (PE), fermentation products (FP), vitamins (V), drugs (D), lipids (L), fungus extracts (FE), polysaccharides (P), acid/salts (AS), blend of essential oils and organic acids (B), yeasts (Y), modified sugars (MS), simple sugars (SS) and emulsifiers (E).

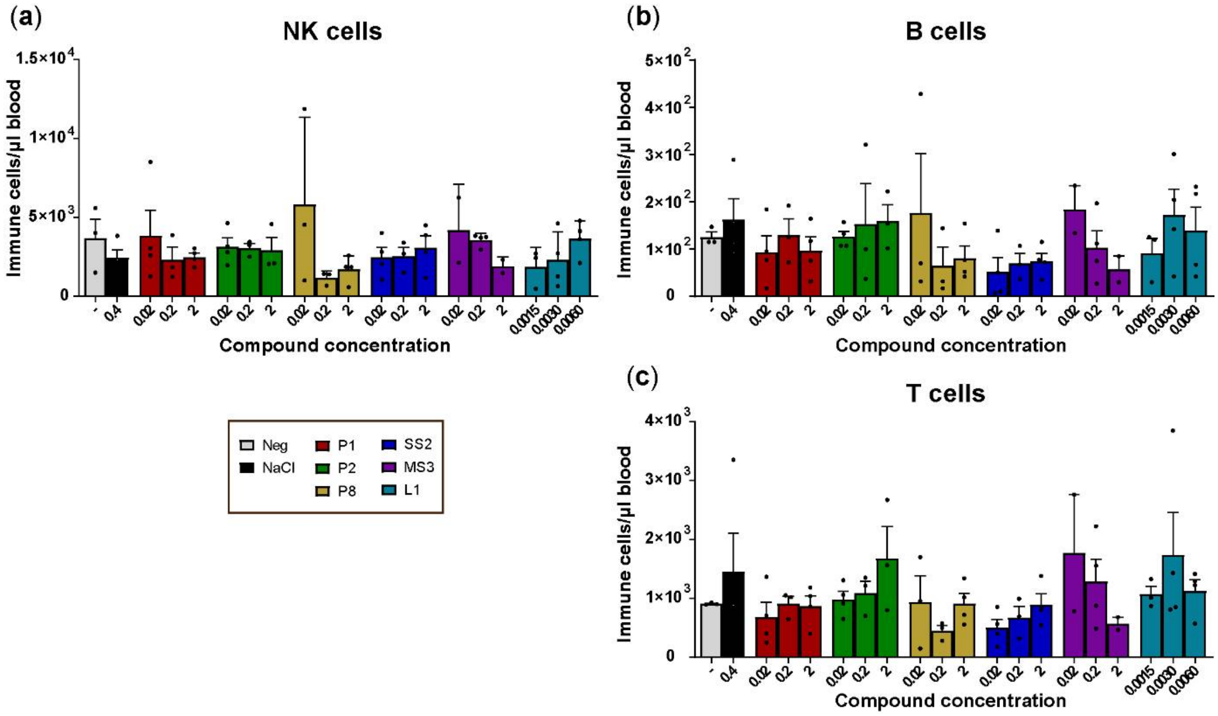

Supplementary Figure S4. In ovo screening of feed compounds for their effect on peripheral immune cell numbers. (**a**) Effect of compound injections in ED18 eggs on numbers of NK cells, (**b**) B cells and (**c**) T cells in whole blood immediately post-hatch. Non-injected eggs were included as negative control and 0.4% saline solution as solvent control. Mean + SEM are shown ($n = 4$) of the compounds polysaccharides (P1, P2, P8), simple sugars (SS2), modified sugars (MS3) and lipids (L1) in different concentrations (0.02, 0.2, 2% or 0.0015, 0.0030, 0.0060mg).

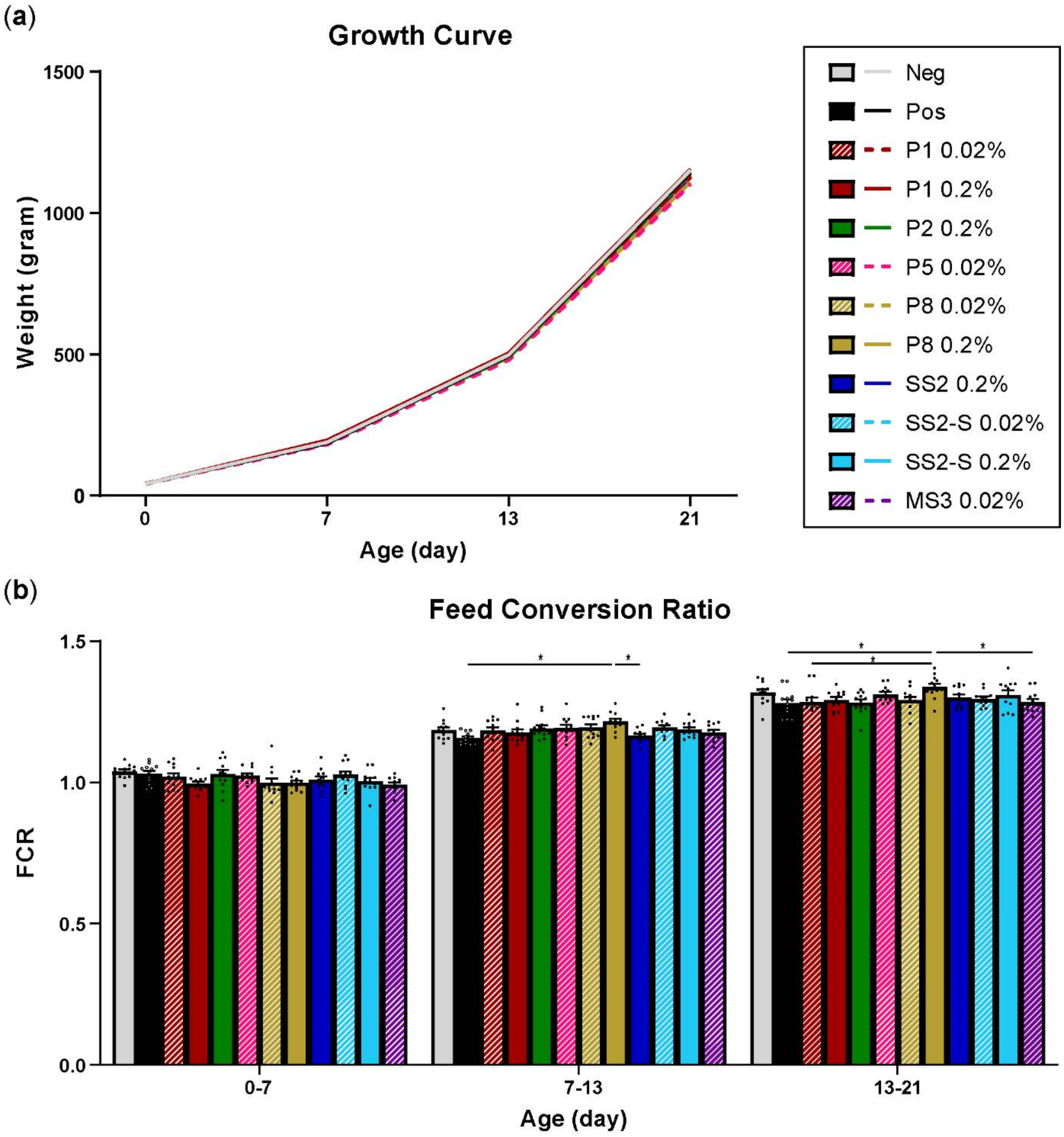

Supplementary Figure S5. In vivo screening of diets for their effect on performance traits in broiler chickens. (**a**) Effect of diet on weights (g) of chickens during aging. (**b**) Effect of diet on feed conversion ratio (FCR) during the periods 0-7, 7-13 and 13-21 days of aging. Mean + SEM is shown (n = 12) of broiler chickens provided standard diet (negative control), feed supplemented with 0.0625% SCFP (positive control), or compounds in different concentrations; P1 (0.02%, 0.2%), P2 (0.2%), P5 (0.02%), P8 (0.02%, 0.2%), SS2 (0.2%), SS2-S (0.02%, 0.2%, Sigma-Aldrich) and MS3 (0.02%), and statistical significance is indicated as * $p < 0.05$.

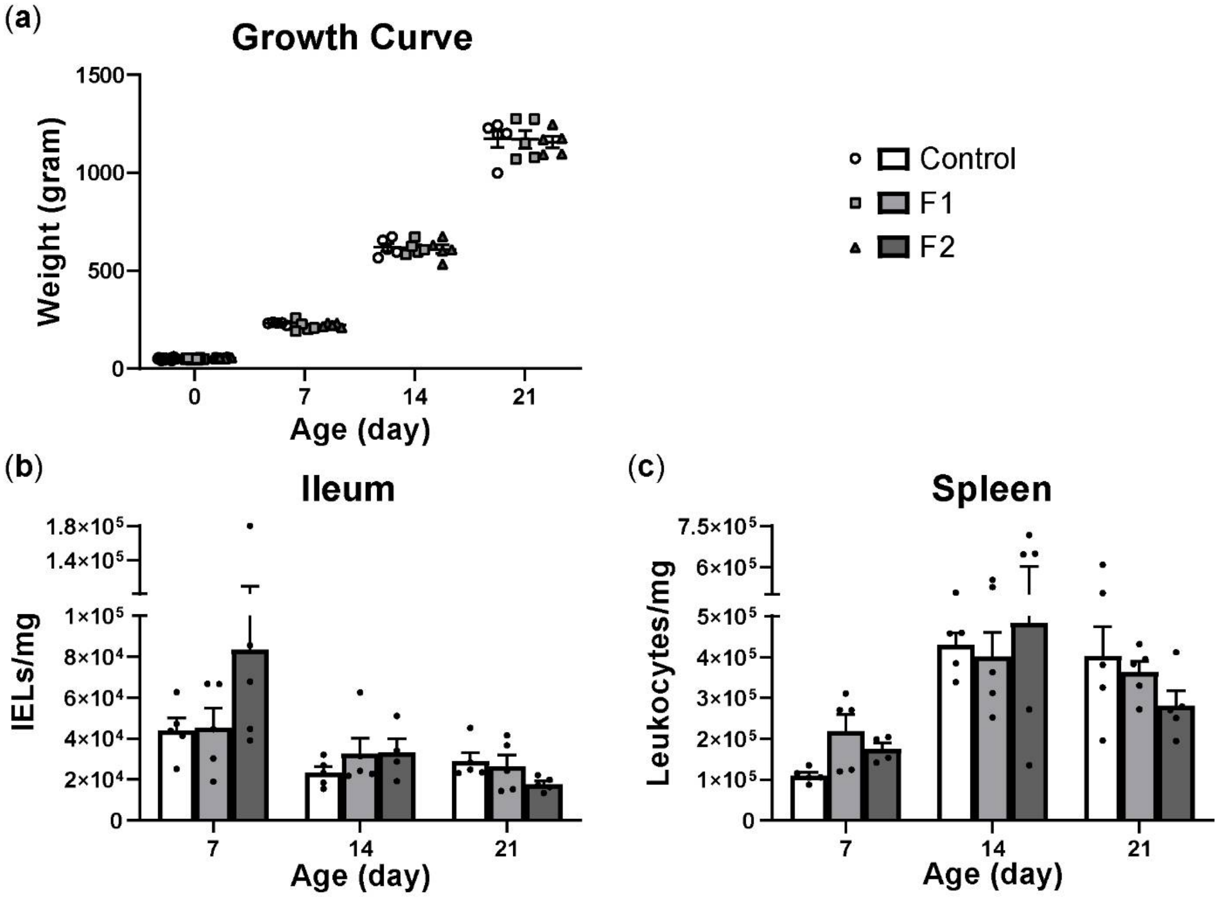

Supplementary Figure S6. Effect of feeding glucose oligosaccharides and long-chain glucomannan to broiler chickens on growth and IELs and splenic leukocytes. (**a**) Weights (g) of chickens provided different diets in the course of time. (**b**) Numbers (cells/mg) of IELs isolated from the ileum and (**c**) splenic leukocytes in broiler chickens provided different diets in course of time. Mean + SEM is shown (n = 5) of chickens provided standard diet (control), feed supplemented with glucose oligosaccharide (F1) or long-chain glucomannan (F2).

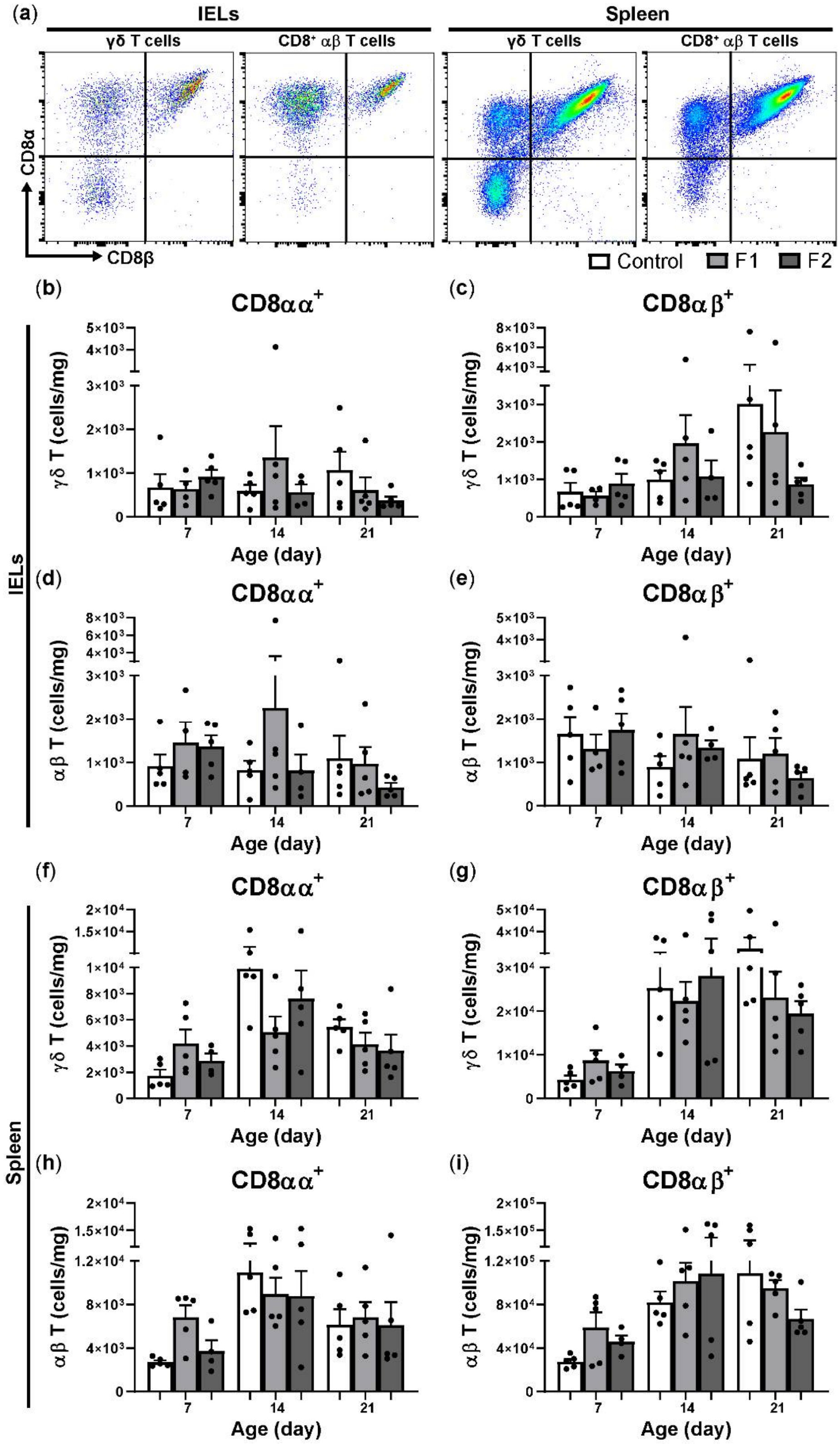

Supplementary Figure S7. Effect of feeding glucose oligosaccharides and long-chain glucomannan to broiler chickens on intraepithelial and splenic γδ T cells and cytotoxic T cells expressing either CD8*αα* or CD8*α*β. (**a**) γδ T cells and CD8⁺ *α*β T cells expressing either CD8*αα* or CD8*α*β in the IEL population (first and second panels) and spleen (third and fourth panels). (**b**) Numbers (cells/mg) of intraepithelial CD8*αα*⁺ γδ T cells, (**c**) CD8*α*β⁺ γδ T cells, (**d**) cytotoxic CD8*αα*⁺ *α*β T cells and (**e**) CD8*α*β⁺ *α*β T cells in broiler chickens provided different diets in the course of time. (**f**) Numbers (cells/mg) of splenic CD8*αα*⁺ γδ T cells, (**g**) CD8*α*β⁺ γδ T cells, (**h**) cytotoxic CD8*αα*⁺ *α*β T cells and (**i**) CD8*α*β⁺ *α*β T cells in broiler chickens provided different diets in the course of time. Mean + SEM is shown (n = 5) of chickens provided standard diet (control), feed supplemented with glucose oligosaccharide (F1) or long-chain glucomannan (F2).

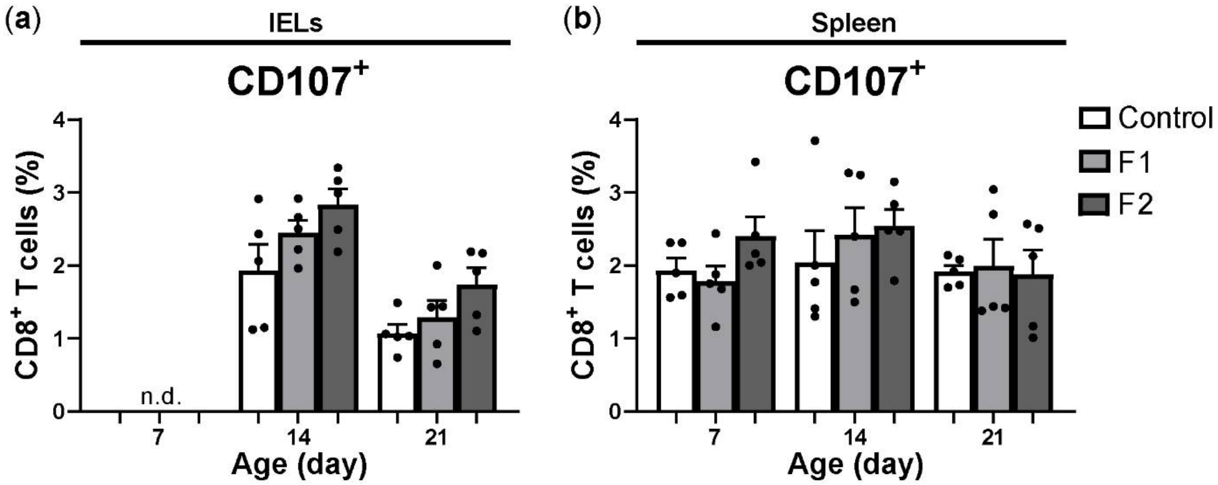

Supplementary Figure S8. Effect of feeding glucose oligosaccharides and long-chain glucomannan to broiler chickens on intraepithelial and splenic T cell activation. (**a**) Percentages of $CD8^+$ T cells expressing CD107 (including both $\gamma\delta$ and $\alpha\beta$ T cells) in the IEL population and (**b**) spleen in broiler chickens provided different diets in the course of time. Mean + SEM is shown (n = 5) of chickens provided standard diet (control), feed supplemented with glucose oligosaccharide (F1) or long-chain glucomannan (F2), for chickens at day 7 in the IEL population percentages were not determined (n.d.) due to numbers of events acquired in the gate of interest were < 100.

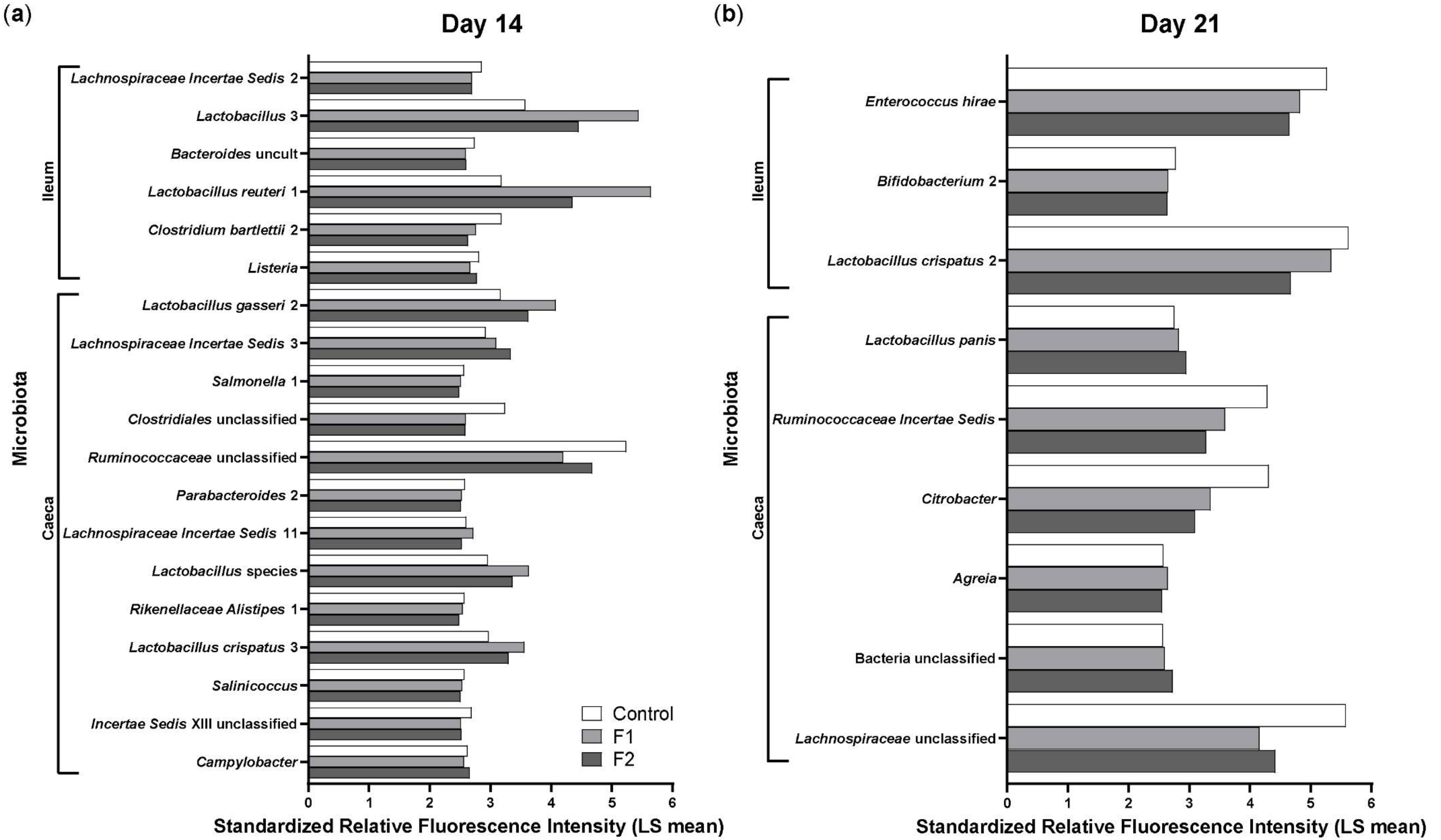

Supplementary Figure S9. Relative abundance of intestinal microbial taxa significantly increased with respective feed at day 14 and 21 in broiler chickens. (**a**) Standardized relative fluorescence intensities (LS means) of the microbial taxa in the ileum and caeca as measured by the microarray (Table 2) that were significantly higher with standard diet (control), feed supplemented with glucose oligosaccharide (F1) or long-chain glucomannan (F2) at day 14 and (**b**) day 21 in broiler chickens. LS mean per microbial target and diet group are shown (n = 5) with statistical significance of FDR adjusted p-values set at < 0.05.

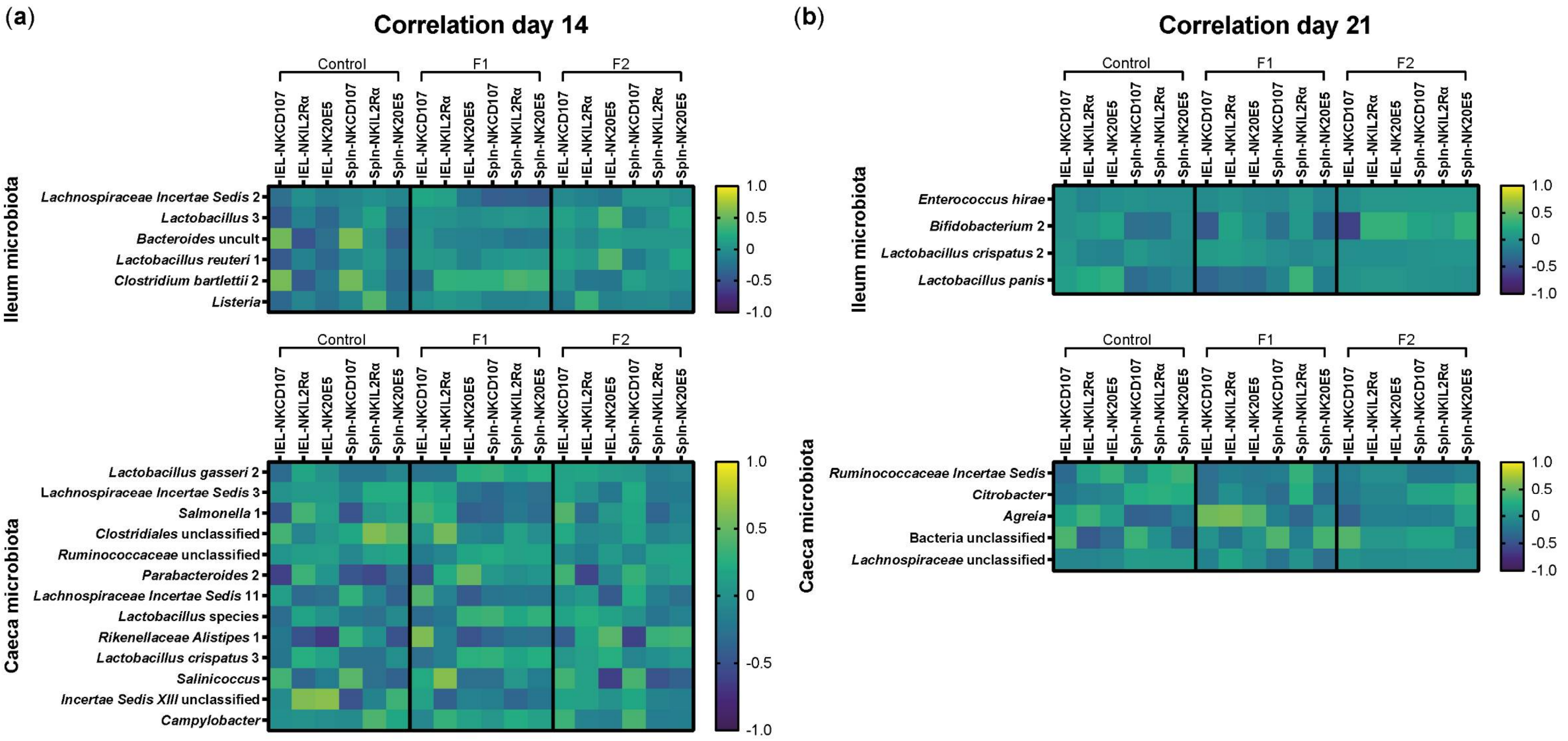

Supplementary Figure S10. Correlations between intestinal microbial taxa and intraepithelial and splenic NK cells at day 14 and 21 in broiler chickens. (**a**) Correlation values between microbial taxa in the ileum (top) and caeca (bottom) significantly increased with the respective feed and percentages of NK cell activation or numbers of NK cell subsets of the ileum (IELs) and spleen (Spln) per diet group (control, F1, F2) at day 14 and (**b**) day 21 in broiler chickens. Pearson's correlation (r) values are depicted in a heatmap as positive (yellow) or negative (dark blue) correlations.
